# The Effect of Dextrose or Protein Ingestion on Circulating Growth Differentiation Factor 15 and Appetite in Older Compared to Younger Women

**DOI:** 10.3390/nu14194066

**Published:** 2022-09-30

**Authors:** Catrin Herpich, Stephanie Lehmann, Bastian Kochlik, Maximilian Kleinert, Susanne Klaus, Ursula Müller-Werdan, Kristina Norman

**Affiliations:** 1 Institute of Nutritional Science, University of Potsdam, 14558 Nuthetal, Germany; 2 Department of Geriatrics and Medical Gerontology, Charité—Universitätsmedizin Berlin, Corporate Member of Freie Universität Berlin and Humboldt-Universität zu Berlin, 13347 Berlin, Germany; 3 Department of Nutrition and Gerontology, German Institute of Human Nutrition Potsdam-Rehbrücke, 14558 Nuthetal, Germany; 4 Muscle Physiology and Metabolism Group, German Institute of Human Nutrition, Potsdam-Rehbrücke, 14558 Nuthetal, Germany; 5 Department of Nutrition, Faculty of Science, Section of Molecular Physiology, Exercise and Sports, University of Copenhagen, 1165 Copenhagen, Denmark; 6 German Center for Diabetes Research (DZD), DIfE, Potsdam-Rehbrücke, 14558 Nuthetal, Germany; 7 Department of Physiology of Energy Metabolism, German Institute of Human Nutrition Potsdam-Rehbrücke, 14558 Nuthetal, Germany; 8 Evangelisches Geriatriezentrum Berlin gGmbH, 13347 Berlin, Germany; 9 German Center for Cardiovascular Research (DZHK), Partner Site Berlin,14558 Berlin, Germany

**Keywords:** aging, anorexia of aging, GDF15, GLP-1, postprandial

## Abstract

Growth differentiation factor 15 (GDF15) is a stress signal that can be induced by protein restriction and is associated with reduced food intake. Anorexia of aging, insufficient protein intake as well as high GDF15 concentrations often occur in older age, but it is unknown whether GDF15 concentrations change acutely after meal ingestion and affect appetite in older individuals. After an overnight fast, appetite was assessed in older (*n* = 20; 73.7 ± 6.30 years) and younger (*n* = 20; 25.7 ± 4.39 years) women with visual analogue scales, and concentrations of circulating GDF15 and glucagon-like peptide-1 (GLP-1) were quantified before and at 1, 2 and 4 h after ingestion of either dextrose (182 kcal) or a mixed protein-rich meal (450 kcal). In response to dextrose ingestion, appetite increased in both older and younger women, whereas GDF15 concentrations increased only in the older group. In older women, appetite response was negatively correlated with the GDF15 response (rho = −0.802, *p* = 0.005). Following high-protein ingestion, appetite increased in younger women, but remained low in the old, while GDF15 concentrations did not change significantly in either age group. GLP-1 concentrations did not differ between age groups or test meals. In summary, acute GDF15 response differed between older and younger women. Associations of postprandial appetite and GDF15 following dextrose ingestion in older women suggest a reduced appetite response when the GDF15 response is high, thus supporting the proposed anorectic effects of high GDF15 concentrations.

## 1. Introduction

Growth differentiation factor 15 (GDF15) is a cellular stress-induced cytokine, and higher circulating concentrations are found in various chronic and acute diseases [1] as well as in older age [2]. The role and effects of higher GDF15 concentrations, during aging in particular, are unclear.

While most cells and tissues are able to secrete GDF15 [3], the expression of the GDF15 receptor, glial cell-derived neurotrophic factor family receptor alpha-like (GFRAL), has been detected only in the brainstem [4]. Recently, GDF15 has been shown to regulate appetite [5] via GDF15-GFRAL signaling [1]. The activation of this signaling axis is associated with conditioned taste aversion [3,6] and the modulation of the vagal sympathetic nervous system, which controls, e.g., gastric emptying [7]. GDF15 expression is sensitive to various nutritional stimuli such as chronic high-fat overfeeding or lysine-deficient diets, which is mediated by the integrated stress response (ISR) [3].

Aging is frequently accompanied by anorexia of aging, which is characterized by reduced appetite as well as lower food intake, and is associated with an increased risk for malnutrition, since the lower energy intake often coincides with insufficient macro- and micronutrient supply, most importantly of protein [8,9]. The etiology is not clear, but mostly likely involves the interplay of several age-related sensory and metabolic changes [8]. Sensory decline, e.g., the loss of taste and sense of smell, can lead to reduced food palatability. Furthermore, various metabolic alterations contribute to overall increased satiety, and therefore lower appetite and food intake, in the old [8]. For example, postprandial hunger, satiety as well as gastric emptying differ in older compared to younger adults after ingestion of a mixed meal [10]. In this context it is interesting that bariatric surgery, which is known to alter satiety and gastric emptying, increases circulating GDF15 in both men and women [11].

To date, it is not known if the high GDF15 concentrations found in older ages affect appetite in humans, and whether there is an age-dependent difference in the GDF15 response to different meals. In addition, it remains to be elucidated if the GDF15 response to meal ingestion affects postprandial appetite. Overall, there are only a few studies addressing the postprandial response of GDF15 in humans [12,13,14]. Therefore, we investigated postprandial circulating GDF15 and its association with postprandial appetite in young and older women.

## 2. Materials and Methods

This is a sub-analysis of a larger study described elsewhere [15]. In brief, community-dwelling older and younger adults were recruited. In order to obtain a significant age gap between the groups, we pre-specified age ranges of 65 to 85 years for the older group and 18 to 35 years for the younger group. In the older group, we did not recruit adults aged above 85 out of ethical considerations, and in the younger group we did not recruit adults over 35 to preclude any early perimenopausal changes. The study was approved by the ethics committee of the University of Potsdam and registered at drks.de as DRKS00017090. All participants signed a written informed consent.

As the number of men was low and sex differences are known [16], men were excluded from this analysis. One younger woman had to be excluded from postprandial analysis since she did not complete the meal challenge. The postprandial GDF15 response was assessed after dextrose (50 g dextrose, total energy content: 182 kcal; *n* = 10 per age group) or high-protein ingestion (77 energy percent protein, total energy content: 450 kcal; 250 g curd cheese, 50 g protein powder, 100 g raspberries, 100 mL milk 1.5% fat; *n* = 10 older group, *n* = 9 younger group). Blood samples were drawn after an overnight fast and repeated blood samples were taken 30, 60, 120 and 240 min after meal ingestion. EDTA plasma was obtained and stored at −80 °C until analysis. Subjective appetite was assessed using a visual analogue scale. Participants were instructed to mark their current feeling of appetite (ranging from 0 = “no appetite” to 10 = “great appetite”) every time a blood sample was drawn. Appetite sensation was displayed in cm on the VAS. Fat mass was estimated using bioelectrical impedance analysis and age-appropriate equations [17]. Fat mass index (FMI) was calculated by dividing the fat mass (kg) by height squared (m^2^).

Plasma GDF15 (intra-assay CV: 6.3–7.2%; inter-assay CV: 2.9–5.6%; BioVendor, Brno, Czech Republic) concentrations were quantified using commercial ELISA assays. As an objective marker for appetite/satiety, we also measured glucagon-like peptide 1 (GLP-1) (intra-assay CV: 4.69–10.7%; inter-assay CV: 9.63–17.6%; Yanaihara Institute Inc, Shizuoka, Japan). As markers of the glucose metabolism, serum insulin (commercial ELISA, intra-assay CV: 4.8–6.0%; inter-assay CV: 8.1–9.0%; BioVendor, Brno, Czech Republic) as well as serum glucose concentrations (colorimetric method, ABX Pentra 400, Horiba, Ltd., Kyoto, Japan) were quantified. Homeostasis model assessment was used to estimate insulin resistance (HOMA-IR).

Statistical analyses were performed with SPSS (IBM version 27, SPSS Incorporated, Chicago, IL, USA) and GraphPad Prism (version 7.00 for Windows, GraphPad Software, La Jolla, CA, USA). Data are presented as mean ± standard deviation (SD). Group differences were calculated using as appropriate Student’s *t*-test or Mann–Whitney U test and correlations with Pearson´s correlation coefficient or Spearman’s rho. GLP-1 concentrations were logarithmized for normalization. Changes over time and time × meal interactions were examined with repeated measures ANOVA. GDF15, GLP-1, glucose and insulin response, and increase in appetite after meal ingestion were evaluated using positive incremental area under the curve (iAUC). An acceptable level of statistical significance was established a priori at *p* < 0.05.

## 3. Results

A description of the participants is shown in Table 1. The study participants were overall healthy, with self-reported high blood pressure being the most frequent pre-existing condition in older women (50%). Despite having higher fasting glucose concentrations, older women exhibited similar insulin and HOMA-IR values to younger women. Baseline GDF15 concentrations were significantly higher in the older compared to the younger women (802 ± 227 versus 364 ± 125 pg/mL, *p* < 0.001). 

**Figure 1:**
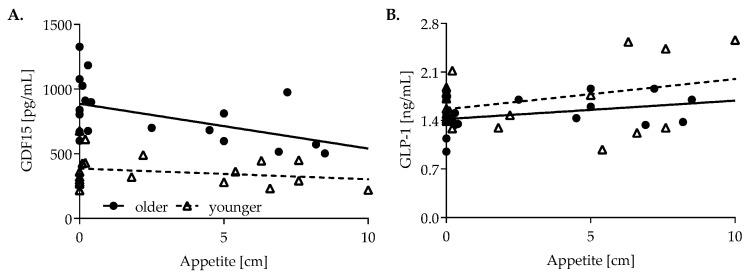
Correlation of baseline appetite and baseline (**A**) GDF15 (older: r = −0.488, *p* = 0.029; younger: r = −0.228, *p* = 0.347) and (**B**) GLP-1 concentrations (older: older, r = 0.461, *p* = 0.041; younger: r = 0.227, *p* = 0.350). GLP-1 was logarithmized for normalization. Correlations of GLP-1 were calculated using log-transformed values but are shown as untransformed values for better visualization. Closed circles represent older women, open triangles younger women.

BMI was similar between both groups, but older women exhibited a higher fat mass (8.65 versus 7.0 kg/m^2^, *p* = 0.036). Fat mass index was also positively correlated with fasting GDF15 concentrations (r = 0.346, *p* = 0.029), but not with BMI (Supplementary Material Figure S1). In older women, baseline GDF15 concentrations were negatively correlated with baseline appetite (r = −0.488, *p* = 0.029), whereas fasting GLP-1 concentrations were positively associated with baseline appetite (r = 0.461, *p* = 0.041) (Figure 1). Glycemic parameters were not correlated with GDF15 or GLP-1.

### 3.1. Appetite

**Figure 2:**
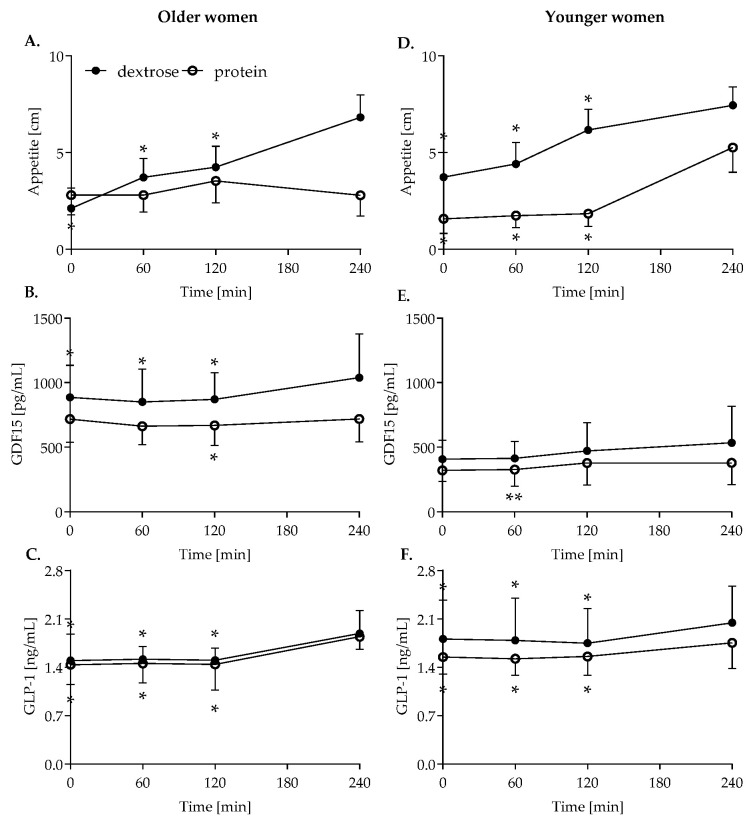
Postprandial appetite (**A**), GDF15 (**B**) and GLP-1 (**C**) concentrations in older and younger women ((**D**,**E**,**F**), respectively) following dextrose (closed circles) or protein (open circles) ingestion. GLP-1 concentrations were logarithmized for normalization. Repeated measures ANOVA, data are shown as mean ± SD. * indicates significant difference to 240 min, ** to 120 min, separately for both test meals. Postprandial changes of GLP-1 concentrations were calculated using log-transformed values but are shown as untransformed values for better visualization. *n* = 10 per group; *n* = 9 in younger high-protein group. Closed circles represent older women, open circles younger women.

Overall, in both older and younger women, appetite changed over time (*p* = 0.015 versus *p* < 0.001; Figure 2A,D), but only in older women did postprandial appetite differ between the two test meals (*p* = 0.015 versus *p* = 0.383 in younger women). Following dextrose ingestion, appetite significantly increased in both age groups during the meal challenge. After protein ingestion, only younger women exhibited increasing appetite from 120 to 240 min, whereas appetite did not change over time in the older women. At the end of the meal challenge, appetite was similar for both test meals in younger women, but in older women appetite was higher after dextrose compared to protein ingestion (mean difference: 4 cm, *p* = 0.021).

### 3.2. GDF15

GDF15 concentrations significantly changed over time in both older (*p* < 0.001) and younger women (*p* = 0.019; Figure 2B,E). Only in older women did the test meal have an effect on postprandial GDF15 concentrations (*p* = 0.026). Following dextrose ingestion, GDF15 concentrations significantly increased in older women from 60 to 240 min, but not in younger women. After protein ingestion, GDF15 concentrations slightly increased in older women from 120 to 240 min and from 60 to 120 min in younger women. However, in older women, GDF15 concentrations were higher in response to dextrose compared to protein at 120 min (mean difference: 202 pg/mL, *p* = 0.023) and 240 min (mean difference: 320 pg/mL, *p* = 0.017) after meal ingestion. GDF15 concentrations during the meal challenge were not different between dextrose or high-protein ingestion in younger women.

### 3.3. GLP-1

In both age groups, GLP-1 concentrations increased after meal ingestion (older: *p* < 0.001, younger *p* < 0.001; Figure 2C,F), but were not different between the meals. 

### 3.4. Glucose Metabolism

**Figure 3:**
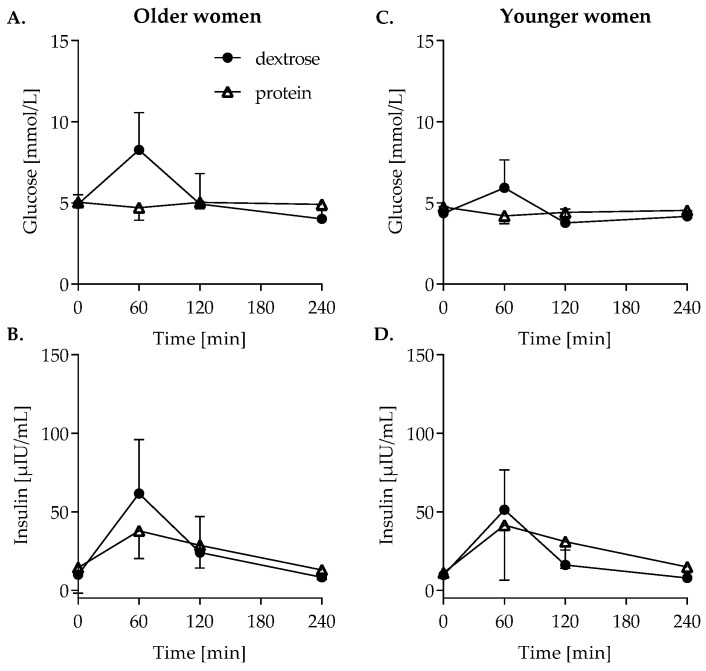
Postprandial glucose (**A**) and insulin (**B**) concentrations in older and younger women ((**C**,**D**) respectively) following dextrose (closed circles) and high protein (open triangles ingestion. Repeated measures ANOVA, data are shown as mean ± SD. *n* = 10 per group; *n* = 9 in the younger high-protein group. Closed circles represent older women, open triangles younger women.

Postprandial glucose and insulin concentrations after meal ingestion are depicted in Figure 3. Glucose concentrations significantly changed over time in both older (*p* < 0.001, Figure 3A) as well as younger women (*p* = 0.012, Figure 3C) and were different between test meals (old: *p* < 0.001, young: *p* = 0.001). Postprandial insulin concentrations also significantly changed over time in both older and younger women (*p* < 0.001 in both age groups), but only in older women, the insulin concentration changes over time were different between the test meals (*p* = 0.027; Figure 3B,D).

### 3.5. Association of Appetite, GDF15, GLP-1, Glucose and Insulin Response 

**Figure 4:**
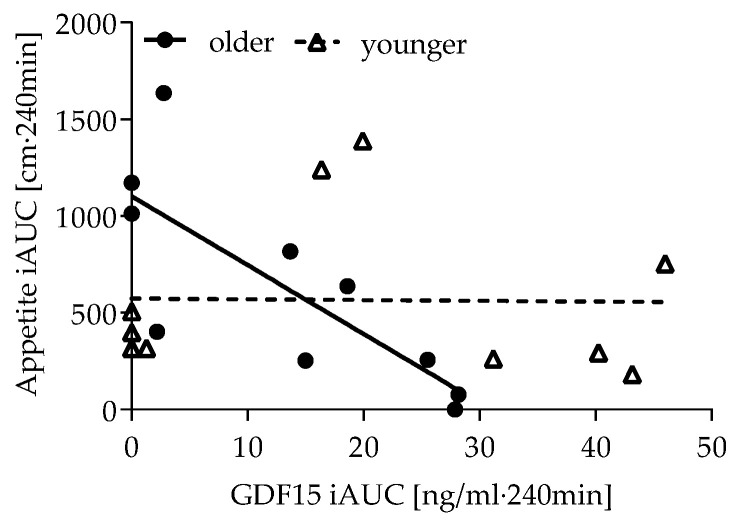
Correlation of appetite iAUC and GDF15 iAUC after dextrose ingestion in older (rho = −0.802, *p* = 0.005) and younger women (rho = −0.215, *p* = 0.550). iAUC: incremental area under the curve. Closed circles represent older women, open triangles younger women.

To evaluate associations among appetite, GDF15 and GLP-1 response, iAUCs were calculated. There was no age difference regarding appetite, GDF15, GLP-1, glucose and insulin iAUCs (Supplementary Material Figure S2). However, following dextrose ingestion in older women only, appetite iAUC was negatively correlated with GDF15 iAUC (rho = −0.802, *p* = 0.005), which suggests that the increase in appetite was less prominent when the postprandial increase in GDF15 was high (Figure 4). In younger women after protein ingestion, GDF15 iAUC was positively correlated with GLP-1 iAUC (rho = 0.729, *p* = 0.026) and insulin (rho = 0.949, *p* < 0.001), but this was not seen in older women. This might indicate that GDF15 behaves similarly to GLP-1 in the young but not in older women.

## 4. Discussion

Due to its many functions, GDF15 has been a target of pharmacological research to treat, e.g., cachexia and diabetes [18]. Moreover, GDF15 has recently gained attention as a potential biomarker for cellular senescence [19] and a key player in the aging process [2], but also as an important regulator of weight homeostasis and appetite [1,20]. To our knowledge, to date there are no studies investigating postprandial appetite and GDF15 concentrations in older adults compared to younger. In this analysis, we show that acute GDF15 response was different between older and younger women and dependent on the type of test meal in older women. Only in the older group and after dextrose ingestion was an increase in GDF15 concentrations found. Postprandial appetite increased over time in both age groups following dextrose ingestion; however, following protein ingestion, appetite remained low in the old, while increasing in the young. In older women, a higher GDF15 response to dextrose was associated with a lower increase in appetite.

The age difference regarding appetite after high protein ingestion might be due to the slower gastric emptying time in older age [10]. In addition, our results imply that GDF15 affects appetite in older women, since fasting GDF15 concentrations as well as their responses were negatively associated with appetite. However, these results also suggest that in younger women, GDF15 does not influence appetite. Studies in mice imply that the anorectic and even nauseating properties of GDF15 unfold only at high/pharmacological concentrations [21,22]. Yet, it is unclear what levels are required in humans for GDF15 to affect energy balance and exert anorexic effects. The association between GDF15 and appetite might therefore be even more prominent in older adults with strongly elevated concentrations, as seen in disease [16].

The regulation of GDF15 secretion is complex as various organs and tissues produce GDF15 [2]. Moreover, multiple stressors and stimuli are able to regulate its expression. One prominent regulator is the ISR, which is induced in response to, among other things, protein restriction [3]. As the dextrose meal was free of protein, the GDF15 increase after dextrose intake might also be interpreted as a response to a lack of protein. This is supported by the observation that GDF15 concentrations after protein ingestion do not change to the same extent as after dextrose intake. Furthermore, the different amounts of calories ingested (180 versus 450 kcal) might also have affected postprandial GDF15 concentrations. However, as short-term overfeeding studies in humans and rodents did not result in altered GDF15 concentrations [3], this effect appears to be negligible. 

To date, only a few studies have investigated post-meal GDF15 concentrations. One study found increasing GDF15 concentrations after an oral glucose tolerance test (75 g dextrose) in younger to middle-aged adults with obesity [13]. In addition, it was shown that the ingestion of a high-carbohydrate or a high-fat mixed meal (protein content 12 E%) did not result in postprandial changes of GDF15. This was also seen in another study using mixed meals (protein content 15 E%) in a younger cohort [14]. Possibly, the protein content of the meals in these studies was sufficient, and therefore GDF15 expression was not induced. However, in none of these studies was postprandial appetite assessed.

Additionally, insulin resistance might play a role in the acute regulation of GDF15. In response to dextrose ingestion, glucose concentrations rose to higher levels at 60 min in older compared to younger women (8.27 versus 5.93 mmol/L), whereas postprandial insulin concentrations were not different between the age groups. This indicates that the older women in this analysis were more insulin-resistant than the younger women, which is a known age-associated effect. GDF15 and insulin iAUC were strongly positively correlated in younger women after protein ingestion, which is in line with the literature, wherein GDF15 was found to regulate insulin secretion [18]. This suggests a complex interplay between nutritional stimuli, GDF15 and insulin, as this is neither seen in response to dextrose nor in older women.

**Figure 5:**
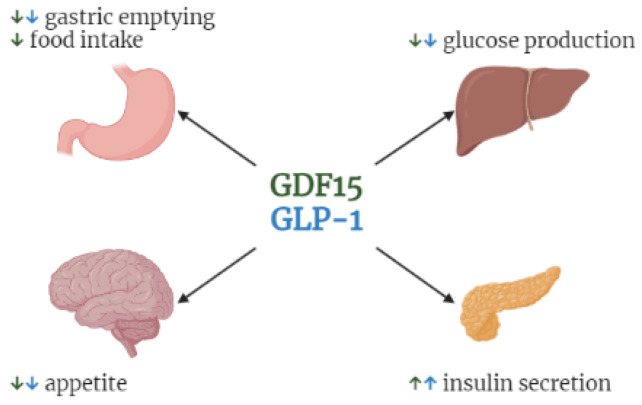
Overview of selected shared functions between GDF-15 and GLP-1. Arrows pointing up indicate enhancing of, arrows pointing down refer to a downregulation. Green refers to GDF15 functions, blue to GLP-1. Created with https://biorender.com/ (accessed on 23 September 2022).

Our study is subject to limitations, such as the number of subjects and the subjective evaluation of the appetite. As a metabolic indicator of energy status as opposed to the subjective rating of appetite, we also analyzed postprandial GLP-1 concentrations. GLP-1 is known to enhance satiety and reduce energy intake [23], and overall exhibits similar actions to GDF15 (Figure 5) [24]. However, GLP-1 is associated differently with fasting and postprandial appetite compared to GDF15. This might imply a dissociation between the expected actions of GLP-1 on satiety and the subjective rating of appetite. A reason for this dissociation might be that humans can be more sensitive to external factors (such as meal size, company while eating, time of day) than internal biological stimuli [25]. Moreover, we were not able to control for all confounders that might have an effect on the findings.

In conclusion, we showed age-specific differences in the appetite’s response to protein intake, and in the GDF15 response following dextrose ingestion. Possibly, studies on subjects with GDF15 concentrations higher than 1200 pg/mL, such as in clinical settings, might reveal a more prominent anorectic effect of GDF15 after meal ingestion.

## Figures and Tables

**Table 1:** Characteristics of study participants at baseline.

	Older Women *n* = 20	Younger Women *n* = 20	*p*-Value
	Mean ± SD	Mean ± SD	
Age (years)	73.7 ± 6.30	25.7 ± 4.39	
BMI (kg/m^2^)	23.9 ± 3.93	22.1 ± 2.33	0.090
FMI (kg/m^2^)	8.65 ± 2.78	7.04 ± 1.73	0.036
Glucose (mmol/L)	4.98 ± 0.44	4.54 ± 0.42	0.003
Insulin (µUI/mL)	12.2 ± 11.5	10.3 ± 2.49	0.488
HOMA-IR	2.70 ± 2.48	2.09 ± 0.58	0.309
GDF15 (pg/mL)	802 ± 227	364 ± 125	<0.001
GLP-1 (ng/mL)	2.60 ± 1.37	3.62 ± 2.29	0.108 ^a^

BMI: body mass index; FMI: fat mass index, GDF15: growth differentiation factor 15; GLP-1: glucagon-like peptide 1; HOMA-IR: Homeostasis Model Assessment—Insulin Resistance; SD: standard deviation, differences between groups calculated using Student’s *t*-test; ^a^ differences between groups calculated using Mann–Whitney U test.

## Data Availability

Data sharing not applicable. Data cannot be shared due to national data protection laws.

## Supplementary Materials

The following supporting information can be downloaded at: https://www.mdpi.com/article/10.3390/nu14194066/s1, Figure S1: Correlation of baseline GDF15 concentrations and (A) FMI and (B) BMI. BMI: body mass index, FMI: fat mass index; Figure S2: Incremental area under the curve (iAUC) for appetite (A), GDF15 (B), GLP-1 (C), glucose (D) and insulin (E) for each test meal and age group. OW: older women, YW: younger women, Dex: dextrose, HP: high protein.
