# Supplementary material for: The Effect of Dextrose or Protein Ingestion on Circulating Growth Differentiation Factor 15 and Appetite in Older Compared to Younger Women
Source: Nutrients. 2022 Sep 30;14(19):4066. doi: 10.3390/nu14194066 (PMC9571024; doi:10.3390/nu14194066)
Supplement: Supplementary file 1 [file nutrients-14-04066-s001.zip › nutrients-1935615-SI.pdf]

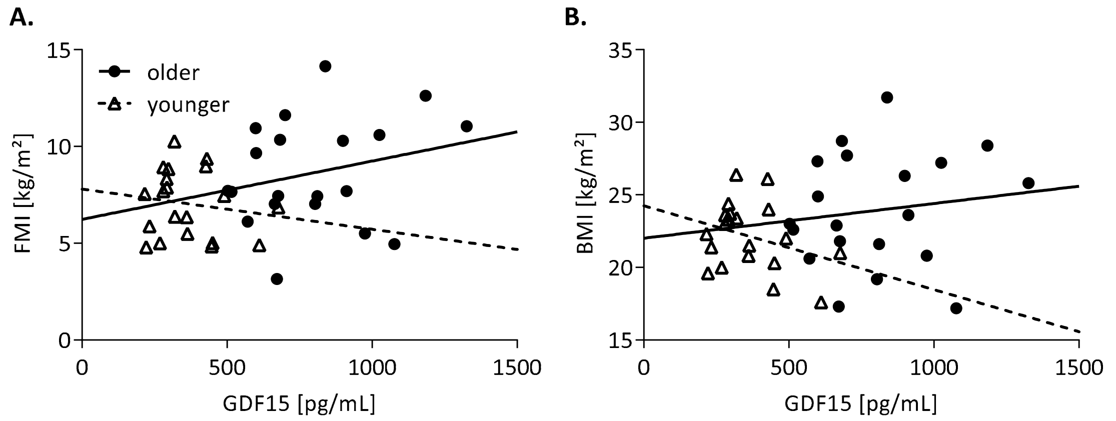

Figure S1. Correlation of baseline GDF15 concentrations and **A.** FMI and **B.** BMI. BMI: body mass index, FMI: fat mass index.

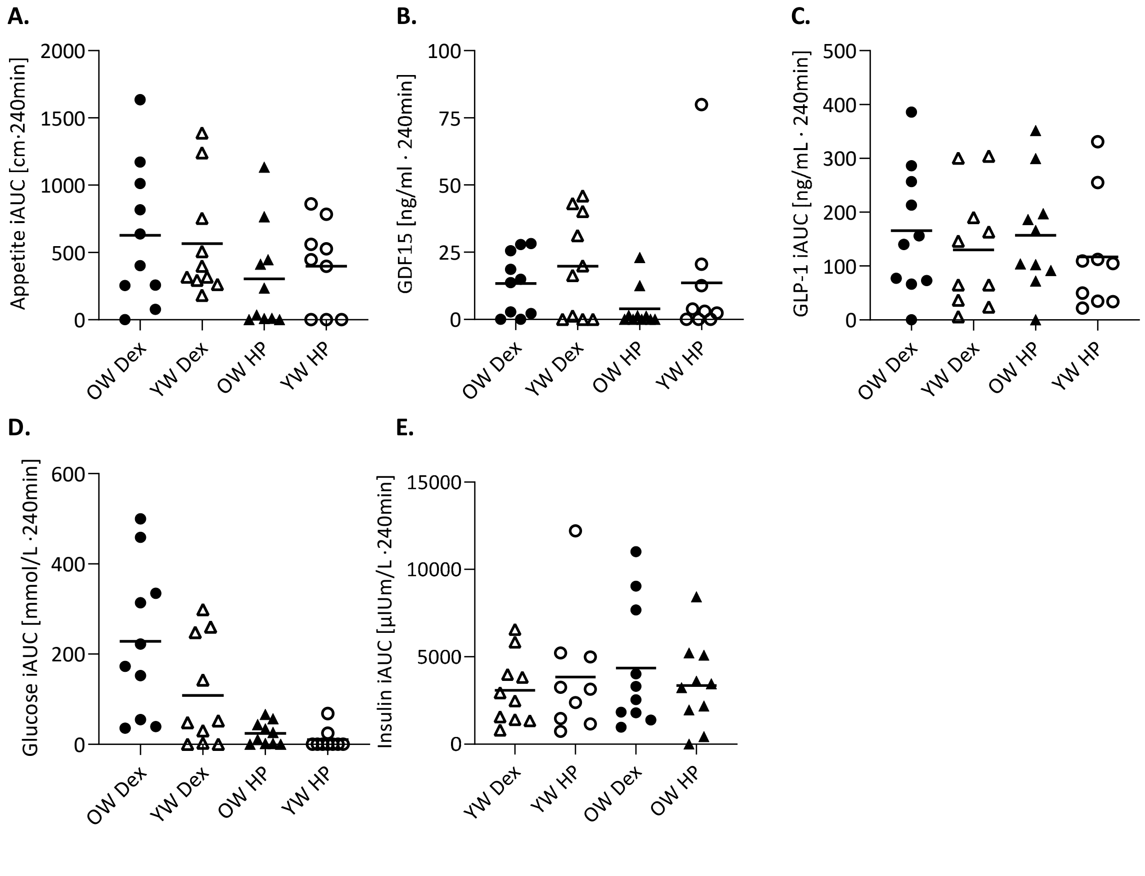

Figure S2. Incremental area under the curve (iAUC) for appetite (A.), GDF15 (B.), GLP-1 (C.), glucose (D.) and insulin (E.) for each test meal and age group. OW: older women, YW: younger women, Dex: Dextrose, HP: high protein.
